# What we are learning on HTLV-1 pathogenesis from animal models

**DOI:** 10.3389/fmicb.2012.00320

**Published:** 2012-08-31

**Authors:** Madeleine Duc Dodon, Julien Villaudy, Louis Gazzolo, Robyn Haines, Michael Lairmore

**Affiliations:** ^1^Laboratoire de Biologie Moléculaire de la Cellule, Unité Mixte de Recherche 5239, Centre National de la Recherche Scientifique, Ecole Normale Supérieure de LyonLyon, France; ^2^UMS3444 Biosciences Lyon-GerlandLyon, France; ^3^Department of Veterinary Biosciences, The Ohio State UniversityColumbus, OH, USA; ^4^Department of Pathology, Microbiology, and Immunology, University of CaliforniaDavis, CA, USA

**Keywords:** animal model, leukemia, immunocompromised mouse, human immune system, retrovirus, HTLV

## Abstract

Isolated and identified more than 30 years ago, human T cell leukemia virus type 1 (HTLV-1) is the etiological agent of adult T cell leukemia/lymphoma, an aggressive lymphoproliferative disease of activated CD4^+^ T cells, and other inflammatory disorders such as HTLV-1-associated myelopathy/tropical spastic paraparesis. A variety of animal models have contributed to the fundamental knowledge of HTLV-1 transmission, pathogenesis, and to the design of novel therapies to treat HTLV-1-associated diseases. Small animal models (rabbits, rats, and mice) as well as large animal models (monkeys) have been utilized to significantly advance characterization of the viral proteins and of virus-infected cells in the early steps of infection, as well as in the development of leukemogenic and immunopathogenic processes. Over the past two decades, the creation of new immunocompromised mouse strains that are robustly reconstituted with a functional human immune system (HIS) after being transplanted with human tissues or progenitor cells has revolutionized the *in vivo* investigation of viral infection and pathogenesis. Recent observations obtained in HTLV-1-infected humanized HIS mice that develop lymphomas provide the opportunity to study the evolution of the proviral clonality in human T cells present in different lymphoid organs. Current progress in the improvement of those humanized models will favor the testing of drugs and the development of targeted therapies against HTLV-1-associated diseases.

## INTRODUCTION

Since antiquity, animals have been part of the human scientific and biomedical landscape. Observational studies of animals as well as experimental investigations using animals have greatly contributed to the advancement of scientific knowledge. Thus, during the last two centuries, biomedical research has largely progressed through observations and experiments performed with a variety of animals, some of which having obtained the status of animal models in biomedical research during the last five decades. Data obtained from *in vivo* biological systems using live animals have progressively completed results from *in vitro* experimental studies. While the great majority of animals are used in the field of pharmaceutical research and toxicology testing, they are also crucial in the understanding of basic and important pathophysiological processes. In particular, mechanisms sustaining the stepwise progression of viral pathogenesis from virus entry to the development of disease has greatly benefited from the use of animal models.

Human T cell leukemia virus type 1 (HTLV-1), the first retrovirus linked to a cancer in humans, was isolated from fresh peripheral blood lymphocytes from patients with cutaneous T cell lymphoma more than 30 years ago. Approximately 20 million people are infected worldwide primarily in endemic regions in Southern Japan, the Caribbean, Western Africa, and Central and South America. Most infected individuals remain asymptomatic for life, but approximately 5% develop adult T cell leukemia/lymphoma (ATL) after decades of protracted viral latency, whereas 1–3% develops inflammatory diseases, such as the neurologic disorder known as HTLV-1-associated myelopathy/tropical spastic paraparesis (HAM/TSP). Clinical features of ATL include monoclonal expansion of leukemic CD4^+^CD25^+^ T cells (with atypical multi-lobulated nuclei) in the peripheral blood. This lymphoproliferative disease in its most aggressive form follows an acute and frequently fatal course with a median 1-year of survival from the time of diagnosis. Patients eventually succumb to clinical complications, in part due to immunodeficiency-induced opportunistic infections.

**FIGURE 1:**
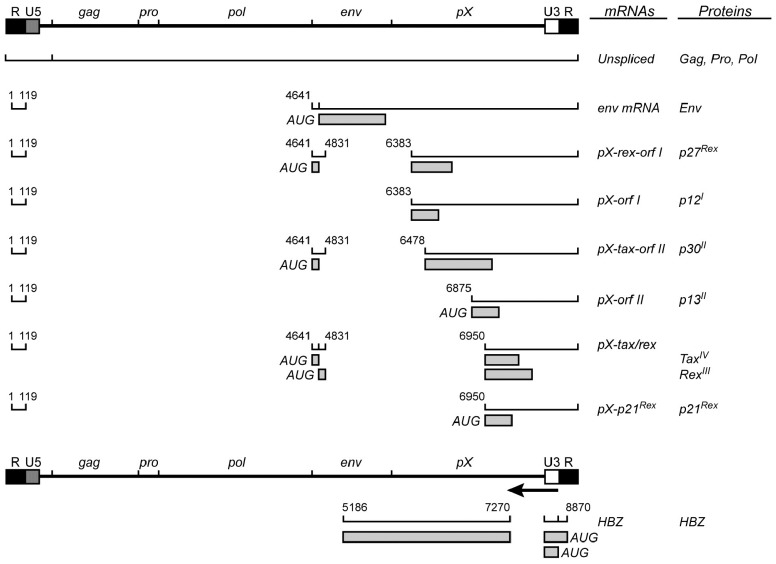
**Human T cell leukemia virus type 1 genome with viral transcripts and encoded proteins**.

Human T cell leukemia virus type 1 is a single-stranded diploid RNA virus that carries genetic information for structural proteins and enzymes (e.g., Gag) and via alternatively spliced mRNAs encodes proteins from open reading frames (ORFs) I–IV (**Figure 1**).

Initial studies on the pathogenesis of ATL have mainly focused on the role of the regulatory Tax (p40) protein, encoded by the ORF IV in the pX proviral region and that behaves as a potent viral and cellular transactivator. Tax promotes proviral transcription and is critically involved in the proliferation of infected T cells, by favoring cell-cycle progression and cell survival and by promoting the activity of cellular transcription factors including NF-κB, AP-1, SRF, and CREB. Interestingly, Tax provokes genomic instability and suppresses host damage response, indicating that Tax harbors oncogenic properties. However, clonal proliferation of ATL cells is frequently associated with a Tax-low or Tax-negative phenotype, as the result of genetic or epigenetic events such as deletion of the 5′ LTR or methylation of this region. Consequently, it is currently accepted that Tax is critically involved in ATL induction and appears to be dispensable in the late stages of this disease. Such an assumption raises the possibility that ATL development and maintenance are dependent on the intervention of another viral product. A few years ago, another regulatory protein named HBZ (HTLV-1 bZip factor) encoded by an ORF in the antisense strand of the HTLV-1 provirus was identified (Mesnard et al., 2006; Duc Dodon et al., 2010). Since this novel viral product has been the subject of intensive research efforts to determine its function in viral replication and/or pathophysiology. Thus, HBZ has been shown to inhibit HTLV-1 transcription by interacting with and displacing CREB from binding to the 5′ LTR and to intervene in the regulation of infected T cells, thus maintaining the leukemic state of these cells.

Other viral products such as the regulatory p27 Rex protein, a product of the ORF III in pX region and the accessory p30 protein (product of ORF II) acting at the transcriptional and post-transcriptional levels have been demonstrated to play a crucial role in the initial stages of infection, mainly by modulating the level of produced viral particles, allowing in turn the promotion of viral persistence and evasion of the host immune surveillance. Likewise, *in vitro* studies have underlined that another auxiliary p12 protein (product of ORF I) that is also promoting viral persistence and immune evasion, has the ability of promote T cell proliferation. Finally the p13 protein (product of ORF II) was found to interact with Tax and suppress its transactivation function and to control the turnover of HTLV-1-infected T cells. In addition, p13, a mitochondria localized protein, influences cell survival (see for review, Lairmore et al., 2011).

It is therefore not surprising that soon after the isolation and characterization of HTLV-1 in 1980, animals were used with the main purpose to uncover the natural history of HTLV-1 infection and of associated diseases in humans. Thus, the use of both small animals (e.g., rats and rabbits) and large animals (e.g., monkeys) has contributed to an understanding of transmission, dissemination, and persistence of HTLV-1 infections. Likewise, induced animal models created by genetic manipulations have been used to delineate the involvement of specific viral genes on the initiation and development of pathophysiological processes in the initiation and the development of HTLV-1-induced pathogenic processes. Furthermore, immunodeficient mice arisen through spontaneous mutations have paved the way not only to the xenograft of leukemic cells, but also to the development of humanized mouse models that have allowed new approaches in translational research.

## ANIMAL MODELS TO STUDY THE EARLY STEPS OF INFECTION

A variety of animal models have been evaluated to study the early events of HTLV-1 transmission and immunologic response to the viral infection. Many of these models have provided important information about viral and host determinants of infection. Rabbits, some non-human primates, and rats have been used to study HTLV-1 transmission and spread, evaluate immune responses against the infection and to test the efficacy of vaccines (Arnold et al., 2006). Rabbits can be consistently infected, but generally do not develop disease, but simulate asymptomatic infections in humans. Cynomolgus macaques (*Macaca fascicularis*) and squirrel monkeys (*Saimiri sciureus*) can be experimentally infected with HTLV-1 and provide useful primate models of HTLV-1 infection (Nakamura et al., 1986; Kazanji, 2000). The monkeys seroconvert, but typically do not demonstrate any clinical signs of disease. Rats have been useful to test immune responses to vaccines and can be experimentally infected with HTLV-1. Because rats exhibit considerable variation in the response to the infection depending on the rat strain used, they are not reliable models to test the early spread of the virus (Kannagi et al., 2000; Hakata et al., 2001).

**FIGURE 2:**
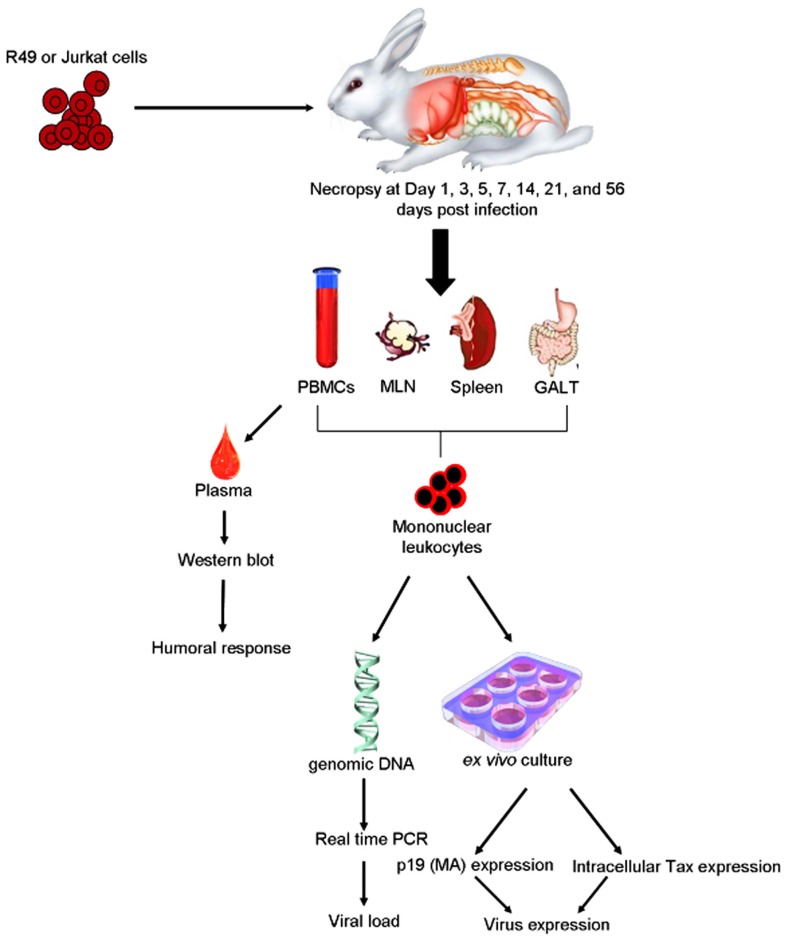
**Example of rabbit model of HTLV-1 transmission**. Inoculation of HTLV-1 transformed cells, e.g., R49 cells or Jurkat cell controls intravenously or orally to establish infection. Viral parameters measured are similar to those used to detect the infection in humans. PBMC, peripheral blood mononuclear cells; MLN, mesenteric lymph nodes; GALT, gut-associated lymphoid tissues.

Rabbits have been used extensively as a model of HTLV-1 infection due to the consistency of transmission, ease of handling, and low cost of maintenance (**Figure 2**).

The rabbit model of HTLV-1 is a reproducible system to produce persistent infections with widespread distribution of the virus similar to humans. The main disadvantage of this model is the lack of spontaneous development of disease. As a model of infection the rabbit has played an indispensable role in evaluation of immune responses, parameters of infection, routes of transmission, and viral genetic determinants of infection with the use of HTLV-1 molecular clones. When compared to the non-human primate model of infection, the rabbit is just as easily infected and offers the advantage of being available in specific pathogen free (SPF) strains. The first studies in the 1980s, showed the ability to infect rabbits using the intravenous route and the MT-2 cell line (a T cell leukemia line derived from an ATL patient; Miyoshi et al., 1985). Early studies with rabbits verified suspected routes of transmission and indicated how antibody responses developed following exposure. These studies also provided important knowledge about the early sequential events following inoculation, and methods to detect proviral DNA in infected tissues. The examination of antibody responses has shown that rabbits respond similarly to humans infected with HTLV-1 and provided opportunities to test immunodominant viral epitopes for use in vaccine approaches using synthetic peptides and recombinant vaccinia vectors. Immunization of rabbits with synthetic peptides verified immunodominant epitopes of the viral envelope protein (Env).

The development of infectious molecular clones in the 1990s provided a unique tool for the evaluation of viral determinants of HTLV-1. One of these clones, ACH, has been widely used to develop stably transformed human and rabbit HTLV-1 positive T cell lines for the use in *in vivo* studies (Collins et al., 1996). Subsequently, ACH clones with mutations within the ORFs encoding the HTLV-1 accessory proteins, p12, p13, and p30, were generated, and used in rabbits to demonstrate the necessity of these accessory proteins for establishment of infection and maintenance of proviral loads. The rabbit model is useful to study the sequential events during early HTLV-1 spread and offers a model to test therapeutic strategies during mucosal transmission. An extensive study on the early spatial and temporal events following intravenous inoculation of HTLV-1 in New Zealand White rabbits indicated proviral reservoirs in lymphoid organs and gut-associated lymphoid tissue (GALT), particularly within intraepithelial lymphocytes (Haynes et al., 2010). This information suggests that the GALT may be a reservoir for infected lymphocytes prior to the development of persistent infections. These data are consistent with a variety of human studies that support emerging evidence that HTLV-1 promotes lymphocyte proliferation preceding early viral spread in lymphoid compartments to establish and maintain persistent infection. Immunosuppressive treatment prior to HTLV-1 infection in the rabbit model (similar to human transplant patients exposed to contaminated blood products) results in enhanced early viral expression.

Infectious clones were also critical to demonstrate the role of pX ORF I and II in the early spread of the virus. Initial *in vitro* studies with molecular clones with pX mutations suggested that pX encoded proteins were not required for infectivity. However, subsequent studies in the rabbit model showed that the proteins encoded in pX ORF I and II were necessary for early establishment of infection and maintenance of proviral loads (reviewed in Lairmore et al., 2011). The first evidence that pX ORF I was important for viral transmission was demonstrated in the rabbit model using splice acceptor site mutant of the ACH infectious molecular clone. The deletion of this p12 acceptor splice site would also introduce a frame shift in the HBZ antisense ORF, resulting in the deletion of the last 24 amino acids of HBZ. However, the replication capacity of subsequent specific HBZ mutants in context of molecular clones did not result in complete reduction of infectivity as observed in ORF I splice mutants. Thus, the rabbit model was able to detect selected HBZ mutations and demonstrated that these were different in viral spread compared to ORF I mutants (Lairmore et al., 2011).

A recent study tested a variety of HTLV-1 mutant molecular clones for their ability to replicate in dendritic cells (DCs) and *in vivo* in rabbits and macaques (Valeri et al., 2010). In this study, rabbits inoculated intravenously with these mutant clones had reduced viral loads at 16 weeks post-inoculation before recovering to “wild type” controls. DC cultures from macaques infected with these mutant clones had reduced viral replication parameters suggesting the importance of this cell type in early viral transmission (Valeri et al., 2010). This study in macaques confirmed the role of proteins encoded in pX ORF I and II in viral transmission.

Collectively, using these animal models we now know that the non-structural genes encoded from ORF I and II are vital for viral infectivity, maintenance of the virus life cycle and proviral loads *in vivo*, as well as host cell activation and regulation of viral gene transcription. Limitations of the rabbit model include the lack of consistent disease that mimic those observed in humans infected with HTLV-1 and the lack of a wide-range of immunologic reagents to assess the model.

## TRANSGENIC AND XENOGRAFT MOUSE MODELS

Mouse models have the distinct advantage of being economical and readily available to test the growth of tumors. They permit studies to test the development of tumors and to test preclinical efficacy of potential therapeutic agents. Multi-centric growth of tumor cells transplanted into immunocompetent mice is concurrent with elevated biomarkers such as PTHrP expression and increasing levels of serum interleukin (IL)-2Rα and β-2 microglobulin that correspond to increasing tumor burden. Even if immunocompetent mice are not efficiently infected with HTLV-1, they have been extensively used to produce animal models exhibiting specific symptoms and pathology of diseases through selective breeding and genetic modification. Thus, transgenic mice have been very useful to test the role of HTLV-1 gene products in the outgrowth of tumors (reviewed in Zimmerman et al., 2010). Furthermore, the use of naturally immunodeficient mice to harbor cancer cells were crucial to the development of mouse xenograft models have been developed to test organ engraftments with ATL cell lines and tumor cells directly derived from ATL patients.

### TRANSGENIC MOUSE MODELS OF HTLV-1

Since the late 1980s, a variety of transgenic mouse models were created to test the role of HTLV-1 gene products in tumor formation or associated disease syndromes. Early models were primarily focused on the Tax oncoprotein. Transgenic mouse models demonstrated the role of Tax in cellular transformation and defined a variety of influences of Tax in cellular signaling pathways such as NF-κB and serum response factor pathways (Nerenberg, 1990). Transgenic mice expressing the HTLV-1 *tax* gene under the control of the LTR promoter developed multicentric mesenchymal tumors of bone, nose, ear, mouth, tail, and foot (Ruddle et al., 1993). This model clearly demonstrated the ability of Tax to elicit tumor formation, despite the fact the model failed to produce lymphomas or leukemia similar to ATL patients. Some of these early transgenic mouse models also developed a wasting disease characterized by degeneration of oxidative muscle fibers, offering insights into the potential role of Tax in HTLV-1-associated myopathies. LTR-tax mice also exhibited skeletal abnormalities and salivary and lacrimal lesions similar to humans suffering from Sjögren syndrome (Green et al., 1989).

By controlling Tax expression using the Lck promoter, lymphoma and leukemia have been produced in transgenic mice (C57BL/6-Tg(Lck-HTLV-1 Tax) (Hasegawa et al., 2006). In this model pre-T cell tumors were associated with constitutive NF-κB activation. Similarly, by limiting expression of Tax to leukocytes transgenic mouse (C57BL/6TgN(huGMZBTax) under the control of the granzyme B promoter exhibit a large granular lymphocytic leukemia and neutrophilic dominated inflammatory lesions (Grossman et al., 1995). This mouse model has recapitulated splenomegaly, lymphadenopathy, and inflammatory masses on appendages. Interestingly, these transgenic mice have humoral hypercalcemia of malignancy (HHM) and osteolytic bone lesions. This model has been extended by testing Tax-mediated activation of luciferase in LTR-luciferase (LTR-LUC) mice (C57BL/6TgN(LtrLuc) for imaging tumor engraftment (Rauch et al., 2009).

Specifically, targeting Tax expression using bitransgenic and doxycycline-inducible mice (Tg(EmuSR-tTa)83Bop) resulted in activation of NF-κB and a fatal dermatologic disease characterized by infiltration of Tax positive T cells into the dermis and epidermis (Kwon et al., 2005). Finally, a number of transgenic C57/CBA mouse strains with tax under the regulatory control of CD-3ε promoter enhancer sequence have developed mesenchymal tumors at wound sites and mammary and salivary adenomas (Hall et al., 1998).

Collectively, transgenic mice have provided reproducible models to test specific gene products role in tumor formation and in targeting models have provided insights into the pathogenesis of leukemia and paraneoplastic lesions associated with HTLV-1 gene products. A primary disadvantage of these models is the lack of viral gene expression in context to an intact proviral genome and lesion development that clearly mimics the pathogenesis of HTLV-1-associated disease of humans.

### XENOGRAFT MOUSE MODELS OF ATL

The possibility for human cells and tissues to be successfully transplanted into mice requires that these mice are compromised in their capacity to reject xenogenic grafts as a consequence of an absence of murine immune response. In 1966, the first immunocompromised mice to be described were the athymic (nude) mice (Flanagan, 1966). Later on formation of both murine mature T and B lymphocytes was shown to be prevented in SCID mice (CB17-*Prkdc*^scid^) bearing the severe combined immunodeficiency the *Prkdc* (protein kinase, DNA activated, catalytic polypeptide) mutation (Bosma et al., 1983). Engraftment of ATL cells and cell lines within these mice is variable depending on which cell line is evaluated. In general studies that use ATL cells obtained from patients and have not been serially passaged are more reliable in tumor outgrowth in this model. Studies in the SCID mice indicated that irradiation or administration of antibodies to abrogate natural killer (NK) cell function was required for successful engraftment of non-leukemic cells lines such as SLB-1 cells (Stewart et al., 1996). These observations have led to a refinement of mouse models with selected genetic defects in innate immune function to further explore the role of specific effector cells in tumor engraftment.

SCID/beige mouse (CB17.B6-*Prkdc*^scid^/*Lyst*^bg^) have defective B and T cell function, NK cell activity, and defective granulocyte properties. These mice have a higher rate of engraftment role of multiple arms of the immune system in tumor engraftment and spread. This tenet was further demonstrated in non-obese diabetic (NOD) mice that lack functional B and T cells, have low NK cell activity, absence of complement activity, and impaired macrophage and antigen presenting cell function. Compared to the SCID mice and the SCID/bg mice, the NOD/SCID mice (NOD.CB17-*Prkdc*^scid^/NCrCrl) are more susceptible to engraftment with the HTLV-1 transformed cell lines (Parrula et al., 2009). Inoculation of MET-1 cells into NOD/SCID mice results in T cell leukemia with multiple organ involvement, which is accompanied by increased serum calcium levels and enhanced expression of receptor activator of nuclear factor κB ligand (RANKL) on leukemic cells, and secretion of PTHrP and IL-6 (Zhang et al., 2003).

The development of the NOD/SCID mice containing a targeted mutation in the beta-2 microglobulin gene (beta 2-m) resulted in mice that lacked NK cell function (Kollet et al., 2000). This strain of mouse offers the advantage of an increase percentage of tumor engraftment and a reduced time to clinical signs compared to NOD/SCID mice. NOD/SCID gamma c null (NOG; NOD/Shi-*scid*/IL-2Rγ^null^) mice are homozygous for the SCID mutation and a targeted disruption of the IL-2Rγ gene mutation. One significant advantage of NOG mice are efficiency to accept transplanted with human cells compare to other mouse models. These mice, in addition to lacking functional B, T, and NK cells, have a severe reduction in interferon γ (IFN-γ) production which allows these mice to be easily transplanted with ATL cells directly from patients to test factors correlative to known paraneoplastic syndromes associated with HTLV-1 infection, such as HHM (Parrula et al., 2009).

## THE USE OF HUMANIZED MICE TO RECAPITULATE HTLV-1 INFECTION

Although xenograft animal models displaying multiple organ engraftments with ATL cell lines or patient samples have been extensively studied, they failed to provide a complete understanding of HTLV-1-associated ATL development. Thus, developing a faithful animal model able to recapitulate the HTLV-1 infection, and to understand the mechanisms of early infection up to the development of associated pathologies is one of the critical goal in HTLV-1 research.

Despite their advantages (small, easy-to handle and low-cost, genetics easily manipulated), mice are not susceptible to several human-specific pathogens, thus restricting their use in experimental and medical research. This limitation has been overcome with the development of humanized mice susceptible to infection by these pathogens. Here, the “humanized mice” are exclusively restricted to severely immunodeficient mice that have been engrafted with functional human cells and tissues. In those animals, the engrafted human cells and tissues carry the same biological functions as they do in the human body. Consequently, the physiological properties and functions of transplanted human tissues and cells can be readily analyzed in the mouse.

Interestingly, during the last decades, constant efforts to generate mouse models allowing an *in vivo* approach of the human immune system (HIS) have been achieved by successful genetic modifications of immunodeficient mice and by improving engraftment procedures. Actually, humanized mouse technology has made rapid progress, and it is now possible to achieve high levels of human chimerism in various organs and tissues, particularly the immune system and the liver (Legrand et al., 2006a, 2009; Shultz et al., 2007; Koyanagi et al., 2008; Manz and Di Santo, 2009; Brehm et al., 2010; Cachat et al., 2012).

### TOWARD THE GENERATION OF MICE WITH A FUNCTIONAL HUMAN IMMUNE SYSTEM

#### Severe immunodeficiency in mice favors the engraftment of a human immune system

The history of humanized mice truly began in 1983 with the pioneering works of McCune and Mosier, who have transferred in SCID mice (CB17-*Prkdc*^scid^) mature human peripheral blood leukocytes (PBL) (hu-PBL-SCID) or transplanted human blood-forming fetal liver cells, fetal bone, fetal thymus (thy), and human fetal liver (liv) in SCID mice (SCID-hu or SCID-hu-thy/liv mice (McCune et al., 1988; Mosier et al., 1988). Even if some level of hematopoietic development has been achieved, these first attempts did not result in the *de novo* formation of a functional human adaptive immune system. Indeed, the majority of human cells engrafted were T cells, with few B cells, myeloid cells, and NK cells. Furthermore, these humanized mice are “leaky,” spontaneously generating murine T and B cells as they age and have high levels of NK activity, both of which prevent efficient/prolonged xeno-engraftment. To note that HIV-1 infection of such humanized mice has yielded valuable data ranging from the fields of *in vivo* pathogenesis to drug efficacy and passive immunity. However, in these humanized mice, HIV-1 infections were often short-term, providing data only on acute infection and no primary adaptive immune response was mounted against HIV-1. Nevertheless, these hemato-lymphoid hu-PBL-SCID and -SCID mouse models have opened the way to extensive studies of the development and function of the human immune and hematopoietic systems and to the generation of mice with a decreased leakiness and with a diminished level of NK cells. Improvements in the available immunodeficient mouse strains were reached with the NOD SCID mice, commonly used because the NOD mutation results in a reduction of NK cell activity (Suwanai et al., 2010). However, a major disadvantage of the NOD strain is a significant incidence of spontaneous thymic lymphomas, which results in a shortened lifespan.

A valuable mouse model called “BLT (for bone marrow, liver, thymus) model” has been described in 2006 as a humanized mouse model able to mount specific adaptive and innate immune responses. These mice were obtained after transplantation of autologous human hematopoietic fetal liver CD34^+^ cells into NOD/SCID mice previously implanted with human fetal thymic and liver tissues (Melkus et al., 2006; Wege et al., 2008). These mice show long-term systemic repopulation with human T and B cells, monocytes, macrophages, and DCs. T cells in these mice generate human MHC class I- and II-restricted adaptive immune responses to Epstein–Barr virus (EBV) infection and are activated by human DCs to mount a potent T cell immune response to superantigens.

Another mouse model was obtained with mice deficient in the recombinase activating genes 1 and 2 (RAG1^– / –^ and RAG2^– / –^, respectively). They do not exhibit leaky production of murine lymphocytes. However, RAG-deficient animals produce normal levels of NK cells, and thus additional mutations are required in order to produce animals better suited for xeno-engraftment studies.

#### The mutation in the common gamma chain receptor

A major technological breakthrough occurred with the creation of RAG1^– / –^, RAG2^– / –^, and NOD–SCID mice that have mutations in the common gamma chain receptor (γ_c_, also referred to as the IL-2 receptor gamma chain) gene. The γ_c_ is a component of the IL-2, IL-4, IL-7, IL-9, IL-15, and IL-21 receptors and is the gene involved in X-linked SCID (Sugamura et al., 1996). Consequently, lack of IL-7 signaling further blocks T and B cell development while lack of IL-15 signaling also prevents maturation and expansion of NK cells (Tassara et al., 1995; Ohbo et al., 1996). Since SCID animals are experiencing leaky production of T and B cells, the γ_c_ mutation is an useful means to further block maturation of these cells. Finally, the inoculation of human CD34^+^ hematopoietic progenitor and stem cells (HP/HSC) either obtained from fetal liver or from umbilical cord blood has resulted in improved, long-term engraftment of human hematopoietic cell types as well as the ability to generate human immune responses. Combinations of the above mutations so far analyzed for HP/HSC engraftment have included such strains as Balb/c RAG2^– / –^γ_c_^– / –^ (referred as to BRG), NOD RAG1^– / –^γ_c_^– / –^ (NRG), NOD–SCID-γ_c_^– / –^ (NSG; Traggiai et al., 2004; Bente et al., 2005; Brehm et al., 2010; Akkina et al., 2011). It should be noted that the methods used to prepare human HP/HSCs and humanized BRG, NRG, and NSG mice are relatively straightforward (Baenziger et al., 2008). When engrafted with HP/HSCs, these immunocompromised strains bearing a γ_c_ mutation have shown to support much higher levels of hemato-lymphoid engraftment than all previous immunodeficient mice, in terms of the production of human immune cells (including T and B cells, monocytes, macrophages, and DCs), of the reconstitution of human cells into various organs, of the duration of engraftment, and of the ability to generate primary human adaptive immune responses (Shultz et al., 2007). Recently, an analysis of the humanization of these three mouse strains has compared engraftment of human HP/HSCs derived from umbilical cord blood following intravenous injection into adult mice and intracardiac and intrahepatic injection into newborn mice (Ito et al., 2008; Brehm et al., 2010). The latter exhibited enhanced engraftment as compared to adult recipients.

#### Genetic background

More interestingly, immunodeficient NOD strains supported enhanced hematopoietic engraftment compared to the Balb/c strain. Indeed, in BRG mice, human B cells develop and survive robustly, but human NK and T cells survive poorly. Thus, the main sign of disturbed human T cell homeostasis is the low T cell frequency in peripheral lymphoid organs. When these mice were complemented either with human IL-7 or human MHC molecules, that both control naive T cell homeostasis, they exhibit enhanced human T cell numbers but only to a limited extent. Likewise, inoculation of BRG mice with human IL-15/IL-15Ra leads to improved but still suboptimal NK cell accumulation. The different cytokines knocked in these models have been reviewed (van Lent et al., 2010; Willinger et al., 2011).

Interestingly, as the NOD genetic background is known for its defective phagocyte activity, it was inferred that the activity of macrophages and other phagocytic cells plays a role in T and NK cell number regulation. Knowing that the phagocyte activity is inhibited by interactions of CD47 with signal regulatory protein alpha (SIRPα: CD172a), two different experimental approaches have led to the demonstration that functional CD47/SIRPα interactions are required for optimal human T and NK cell homeostasis *in vivo*. The first experimental approach consisted in the introduction of CD47/SIRPα interactions in BRG HIS mice by transducing mouse CD47 into HP/HSC (Legrand et al., 2011). The second experimental approach consisted in the generation of mice that express SIRPα in BRG mice (Legrand et al., 2011; Strowig et al., 2011). Both procedures resulted in an important and selective improvement of HP/HSCs engraftment and of human T and NK cell homeostasis. Even if a moderate increase of B cells was observed, total plasma IgG and IgM concentrations significantly increased. Finally, an improved functionality of the HIS was revealed in these mice, which are comparable to NSG mice. Clearly, CD47/SIRPα interaction appears as a major determinant of escape from phagocyte-mediated cell clearance.

Whatsoever, these current humanized mouse models cannot develop a robust adaptive immune response. Indeed these mice do not express HLA molecules on thymic epithelial cells and human T cells lack the ability to recognize antigen in an HLA-restricted manner. To achieve that HLA restriction *in vivo*, an immunodeficient NSG-HLA-A2 strain was created, by backcrossing the HLA class I transgene onto the NSG background (Strowig et al., 2009; Shultz et al., 2010). Transplantation of purified human HP/HSCs into NSG-HLA-A2 newborns resulted in functional CD4^+^ and CD8^+^ T cells expressing cytotoxic molecules and generating cytokines *in vivo*. Furthermore, these humanized NSG-HLA-A2 mice demonstrated functional HLA-restricted CTL in *in vivo* EBV or Dengue virus infection models (Jaiswal et al., 2009; Shultz et al., 2010).

To date, the newer humanized mouse models have been examined for infection by a variety of human viruses and pathogens including the retroviruses HTLV-1 and HIV-1, the herpes viruses EBV, Dengue virus, hepatitis B- and C-virus, as summarized in **Table 1**.

**Table 1:** Infectious agents studied in humanized mice.

Pathogen	Associated disease	Mouse model	Reference
**Viruses**			
HIV-1	AIDS	BRG	Baenziger et al. (2006)
		NSG	Watanabe et al. (2007a)
		NOG	Watanabe et al. (2007b)
		NOD/SCID	Brainard et al. (2009)
		BLT	
		Rag1^– / –^γ_c_^– / –^	Akkina et al. (2011)
HTLV	ATL, HAM/TSP	NOD/SCID	Banerjee et al. (2010b)
		BRG	Villaudy et al. (2011b)
		NSG	Fujisawa et al. (2011)
EBV	Burkitt lymphoma	BRG	Traggiai et al. (2004)
		NOG	Yajima et al. (2008)
		NSG	Strowig et al. (2009)
		NOD/SCID	Islas-Ohlmayer et al. (2004)
		NOD/SCID BLT	Melkus et al. (2006)
KSHV	Kaposi sarcoma	NOD/SCID	Wu et al. (2006)
Dengue	Dengue	BRG	Kuruvilla et al. (2007)
virus		NOD/SCID	Bente et al. (2005)
HSV-2	Genital herpes	BRG	Kwant-Mitchell et al. (2009)
HCV	Hepatitis C	BRG Fah^– / –^	Bissig et al. (2010)
HBV	Hepatitis B	BRG Fah^– / –^	Bissig et al. (2010)
		BRH uPa^– / –^	Dandri et al. (2001)
CMV	Cytomegalovirus	NSG	Smith et al. (2010)
**Bacteria**			
*Salmonella*	Typhoid fever	BRG	Traggiai et al. (2004)
typhi			

The humanized HIS mice are now used as *in vivo* models in both basic and preclinical research fields. Clearly, humanized mice represent a potent translational model for studying human hematopoiesis, immunity, gene therapy, infectious diseases, cancer, and regenerative medicine without putting patients at risk.

### ESTABLISHING HIS MICE AS A MODEL TO STUDY HTLV-1 INFECTION AND PATHOGENESIS

#### Human T cell development in his mice

**FIGURE 3:**
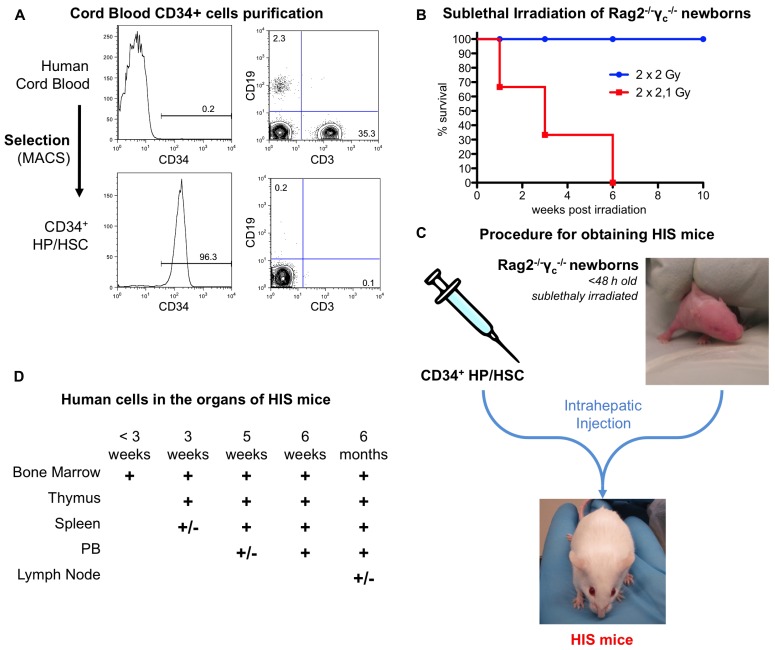
**Generation of HIS mice from human cord blood and Rag2^– / –^γ_c_^– / –^ newborns. (A)** Representative FACS analysis for CD34, CD3, and CD19. CD34^+^ cells are obtained by MACS selection from human cord blood (upper panel). After purification, the purity is over 90% and the B and T cells contamination (lower than 1%, lower panel). The percentage of CD34^+^ cells as well as B (CD19^+^) and T (CD3^+^) cells are indicated on the plots. **(B)** Rag2^– / –^γ_c_^– / –^ newborn mice are sublethally irradiated. **(C)** Schematic representation of the procedure. Purified CD34^+^ cells are injected into the liver of sublethally irradiated Rag2^– / –^γ_c_^– / –^ newborns. This results in the development of a human adaptive immune system in the HIS mice. **(D)** Sequential colonization of the different murine organs by human cells. The humanization can be observed for several months after birth. +/-, less than 5%; +, 5–20%.

Human immune system mice are generated according to the procedure previously described (Traggiai et al., 2004) and summarized in **Figure 3**.

An efficient intrathymic *de novo* development of human T cells is a major prerequisite for humanized mice to be retained as convenient models for human T lymphotropic viruses, such as HIV-1 and HTLV-1. The thymus of newborns HIS (BRG) or HIS (NSG) mice supports human αβT and γδT cell development more efficiently than that of adults, probably because of the involution of the thymic rudiment in the latter (Legrand et al., 2006b). Whatsoever, the establishment of cortex and medulla-like regions are observed within the thymic lobules of the murine thymus. Thymopoiesis occurs independently of the genetic background, in a sustained manner, as indicated by the presence of the four main thymocyte subsets. Thus, in 8 week-old mice, the proportion of double-negative (DN) thymocytes was in the range of 1–2%, while that of double-positive (DP) CD4^+^CD8^+^ thymocytes range from 50 to 80%. Fairly physiological ratios of mature single-positive (SP) CD4^+^ and CD8^+^ T cells are present with broad Vβ distribution, together with *Foxp3*^+^, CD25 regulatory T cells. Furthermore human CD16^+^CD56^+^ NK cells are also observed in the murine thymus.

Mature T cells exit the thymus seeding the peripheral lymphoid organs 1–2 months after inoculation of CD34^+^ cells. They home to bone marrow, spleen, liver, lungs, and peripheral blood (5–20% of human cells). Human T cells developing in HIS mice are functional as indicated by the proliferation of these T cells after *in vitro* stimulation with allogeneic human DCs (Traggiai et al., 2004). The observation that human T cells are able to populate the peripheral lymphoid organs of HIS mice indicates that these cells have undergone a positive selection, potentially through both human and murine MHC molecules.

Beside the development of T cells, other important lineages develop from the human progenitors in HIS mice. The major hematopoiesis-derived cells in HIS mice are B cells undergoing a complete differentiation and maturation process. A low frequency of CD56^+^ NK cells, CD14^+^ monocytes, and conventional and plasmacytoid DCs were also reported in various analyzed organs (Traggiai et al., 2004; Chicha et al., 2005).

Overall, one of the major point achieved by HIS mice is the efficient intrathymic *de novo* development of human T cells, that can be easily studied to answer questions linked to retroviral infection (Baenziger et al., 2008).

#### HIS mice infected with HTLV-1

The first observations of HTLV-1 infections that established humanized mice as *in vivo* models to characterize HTLV-1 tropism and leukemogenesis associated with HTLV-1 infection have been performed in 1996 (Feuer et al., 1996). In that study, CD34^+^ HP/HSCs isolated from human fetal liver cells were productively infected by *in vitro* cocultivation with HTLV-1 producing cell lines. They were then inoculated in SCID mice engrafted with human fetal thymus and liver tissues (SCID-hu-thy/liv). These mice develop a conjoint organ, which supports human thymocyte differentiation and maturation. Viral infection of thymocytes was detected 4–6 weeks later. Interestingly, thymocytes from two mice with the greatest HTLV-1 proviral burdens showed increased expression of the CD25 marker (the IL-2 receptor alpha chain) and perturbation of distribution profiles of the CD4^+^ and CD8^+^ thymocyte subsets. Even if no T cell malignancy was reported, these observations clearly implicate the human thymus as a target reservoir for HTLV-1 infection. They further suggest that the dysregulation of thymopoiesis and lymphoproliferation are predisposing events in the development of the leukemogenic process associated with HTLV-1 infection. It was recently reported that sublethally irradiated 4- to 6-week NOD/SCID mice inoculated with human fetal liver-derived CD34^+^ cells *ex vivo* infected by HTLV-1 developed CD4^+^ T cell lymphomas with characteristics similar to ATL (Banerjee et al., 2010a). Interestingly, an elevated proliferation of infected human stem CD34^+^CD38^-^ was observed in the bone marrow of these mice. Likewise NOD/SCID mice reconstituted with CD34^+^ HP/HSCs transduced with a lentivirus vector expressing the Tax protein also developed CD4^+^ lymphomas. These observations infer that HP/HSCs provide an *in vivo* viral reservoir and act as cellular targets for leukemogenesis in humans.

As underlined above, intensive efforts in the engineering mice strains propitious to humanization have led to the generation of severely immunocompromised strains able to sustain prolonged human hematopoiesis *in vivo* with a higher level of engraftment upon transplantation of CD34^+^ HP/HSCs purified from human cord blood (see above section). The high ability of humanized BRG and NSG mice to support maturation and all human hematopoietic lineages should therefore facilitate the evaluation of the effects of HTLV-1 infection. Thus, it has been reported that in NSG mice inoculated with human fetal liver-derived CD34^+^ cells *ex vivo* infected by HTLV-1, viral infection was skewing hematopoiesis to the T cell lineage leading to hyperproliferation of CD3^+^ T cells in the bone marrow, thymus, spleen, and mesenteric lymph nodes (Banerjee et al., 2010a).

The *ex vivo* infection protocol represent a definite bias as those cells are not naturally *in vivo* infected. Inoculating *ex vivo* HTLV-1 infected CD34^+^ HP/HSC in immunodeficient mice is confusing the humanization process with the infection procedure, which is quite different of the natural infection conditions.

To mimic the conditions favoring the initiation of the leukemogenic process at an early stage of T cell development, it was therefore more suitable to infect mice that were previously humanized. According to that experimental strategy, humanized mice established by the intrabone marrow transplantation of NSG mice with CD133^+^ HSCs purified from human cord blood were infected with HTLV-1 *in vivo* by γ-ray-irradiated HTLV-1-producing T cells, 3–4 months after transplantation (Fujisawa et al., 2011). It was reported that HTLV-1 infection increased the number of CD25^+^CD4^+^ T cells and resulted in splenomegaly. Interestingly, during the late period of infection, when almost all the cells were infected T lymphocytes, the presence of highly lobulated or flower-shaped nuclei appeared in the peripheral blood.

In another study, newborn BRG mice that were intrahepatically inoculated with CD34^+^ HP/HSC isolated from human cord blood were intraperitoneally injected with γ-ray-irradiated HTLV-1-producing T cells, 6–8 weeks after transplantation, at a time when the three main subpopulations of human thymocytes could be detected (Villaudy et al., 2011b). In control animals, i.e., in non-humanized BRG mice infected with HTLV-1, the proviral sequences are not detected. Conversely, the provirus sequences are readily integrated in the human genome in cells from peripheral organs of infected humanized BRG mice up to 8 months after infection. This persistent infection was correlated with an overtime increase of the PVL. Under these conditions, HTLV-1 is inducing alterations of human thymopoiesis, that correlates with level of the proviral load, with an expansion of mature CD4^+^/CD25^+^ T cells and with the development of pathological features, such as splenomegaly and lymphoma phenotypically similar to those found in ATL patients.

Interestingly, these pathologies are associated with a profound deregulation of the T cell development in the infected animals and resulted in the accumulation of infected T cells with a mature phenotype (CD4^+^ or CD8^+^). In this *in vivo* model, genes involved in the anti-apoptotic pathway are deregulated in a manner similar to that observed in *in vitro* Tax-transduced human thymocytes (Wencker et al., 2007, 2009).

#### HTLV-1 infects human immature thymocytes

**FIGURE 4:**
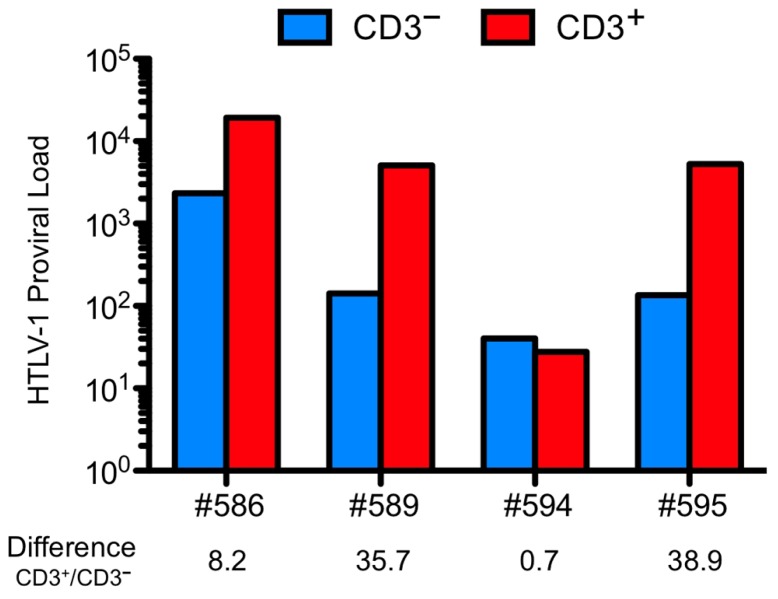
**Human T cell leukemia virus type 1 (HTLV-1) provirus is detected in immature thymocytes and higher PVL is found in more mature thymocytes**. The cells isolated from the thymus of four animals infected at the age of 4 weeks and sacrificed at 18 weeks (#594 and #595) or 31 weeks (# 586 and #589) were sorted on their expression of CD3. Proviral load (PVL) was measured on the DNA extracted form the two sorted populations. Except for the mouse #594, the PVL in the CD3^+^ populations is higher. PVL is defined as the number of copies of HTLV-1 in 100,000 human cells.

To determine in which cells the HTLV-1 provirus is integrated, CD3^+^ cells and CD3^–^ cells were isolated from human CD45-expressing thymocytes of infected HIS mice (**Figure 4**).

The CD3^–^ thymocytes represent immature thymocytes: the early progenitors, the immature CD4 SP population and the early DP T cells. The CD3^+^ thymocytes encompass a mature population with the late DP thymocytes along with SP CD4^+^ or CD8^+^ cells. The level of proviral load in both populations was evaluated and was found to be about 40-fold higher in the CD3^+^ than that in the CD3^–^ population. These observations indicate that immature cells are indeed infected. One important consequence of that event may explain that in the thymus of infected HIS mice, the mature T lymphocytes represent the main subset when compared to the DP and immature subsets. Indeed, no modification in the distribution profiles of these populations has been observed in mock-infected HIS mice (**Table 2**). These observations indicate that the accumulation of mature lymphocytes in the thymus of infected mice is consecutive to their increased capacity of proliferation.

**Table 2:** The human T cell development is altered in HTLV-1 infected HIS Rag2^– / –^γc^– / –^ mice.

	Immature	DP	SP
**Percent of human thymocytes in mock-infected animals**			
Early	18 (3–23)	45 (30–70)	38 (25–60)
Late	10 (5–20)	45 (35–60)	38 (26–58)
Percent of human thymocytes in HTLV-1-infected animals			
Low PVL	20 (2–38)	58 (40–80)	20 (10–40)
High PVL	2 (0–4)	15 (0–38)	75 (50–95)

*Composite data from mock-infected mice and from HTLV-1-infected mice sacrificed early (early and low PVL, respectively) and late (late and high PVL, respectively) after CD34^+^ cell transplantation. Data represent mean ± SD of percent of human CD45^+^ thymocytes that are CD4^–^CD8^–^ and CD4ISP immature cells, DP cells, and SP cells in the thymus of mice.*

Consequently, the thymopoiesis alterations consecutive to HTLV-1 infection may be caused by the expression of Tax in immature thymocytes. Indeed, that viral protein has been shown to down-regulate the expression of the pTα gene, thus perturbing β-selection, the early and critical checkpoint of human αβT cell development in the thymus (Wencker et al., 2007, 2009). Furthermore, the expression of Tax may be linked to the sustained expression of NF-κB-dependent genes that may propel infected thymocytes toward the mature stages of T cell development.

Using a highly sensitive high throughput sequencing system described elsewhere (Gillet et al., 2011), the number of unique integration sites (UIS) as well as the respective size of each clone detected in the spleen of several animals have indicated a polyclonal integration pattern. Interestingly, the analysis of the clonality of different organs in one mouse indicated major differences in the clonal distribution with a high number of UIS in the thymus of this mouse whereas the number of clones in the spleen and the lymph nodes was lower and some clones were quite large in comparison to the others.

### FUTURE PROSPECTS

Thanks to the successive modifications of the model systems and genetic manipulation, humanized mice have acquired the ability to serve as preclinical models for the *in vivo* study of human cells and tissues. Humanized mice could provide valuable information about the cellular and molecular *in vivo* mechanisms supporting the leukemogenic activity of HTLV-1. In addition, they are potentially very helpful for the evaluation of therapeutic treatments.

As suggested in this manuscript, the infected HIS mouse model represents a promising preclinical tool to study new therapeutic treatments. Therapies could be evaluated in collaboration with clinicians to interfere at different times after infection. Treatment to block the entry or the replication of the virus could be assessed in order to serve as a post-exposure way to prevent the persistent infection. A treatment, involving the use of IFN-α, zidovudine (AZT), and As_2_O_3_ has been tested on several patients and has shown promising results as a first-line therapy for ATL patients (Bazarbachi et al., 2010). The molecular and cellular mechanisms involved in this treatment are not yet understood and should benefit from the humanized mouse model. An attempt to elucidate the exact mechanisms was described (Villaudy et al., 2011a).

Intraperitoneal inoculation of HTLV-1-producing cells have been successfully used to infect HIS mice with other human viruses like HIV and represent the easiest way to administrate infected cells into the mice. Oral inoculation is more relevant to the route of natural HTLV-1 infection occurring through breast-feeding, as ATL has been linked to the infection of newborn mostly via breast-feeding by an HTLV-1 infected mother (Hasegawa et al., 2003).

The use of molecular clones to infect the HIS mouse model would represent a powerful tool to dissect the role of the different viral proteins and particularly the regulatory proteins such as Tax, HBZ, Rex, and p30 in an *in vivo* infection and pathogenic processes. This model also represents a good opportunity of understanding the leukemogenic potential of HTLV-1 in a comparative study with HTLV-2 and HTLV-3 which are closely related to HTLV-1 but have different pathogenesis. HTLV-2 has not been associated with any malignant diseases. The pathogenesis of HTLV-3 is unknown due to the low number of known infected people around the world. The infection of HIS mice with HTLV-2 could provide an interesting approach to determine what are the critical steps of leukemogenesis that are created by HTLV-1 and not by HTLV-2 or HTLV-3.

Humanized mice are mainly instrumental in dissecting the early stages of the infection and the critical initial events leading to the development of ATL-like pathologies. It should be emphasized that the proliferation of infected clones is not under immune control, as the human adaptive immune response in this humanized mouse model directed toward HTLV-1 has been very disappointing thus far (Villaudy and Duc Dodon, unpublished observations). The use of alternative immunosuppressed mice strains, such as the NSG-HLA-A2 mice could resolve this problem and improve this model, providing researchers and clinicians with a valuable tool not only to investigate the early steps of leukemogenesis, but also to unravel what is happening during the long latent period following HTLV-1 infection. Over the past three decades, the technology and applications of humanized mouse model have considerably grown providing new opportunity for human retroviral studies and for testing the efficacy of anti-retroviral therapies. The recent development of optimized humanized mice offer great promise in the field of retrovirology knowledge, and reasonable hopes for the development of therapies against this virus-associated cancer.

## CONCLUDING REMARKS

A better knowledge of viral pathogenesis is required to provide comprehensive novel treatments (therapies and vaccines) to eliminate viral infections in humans. Clearly, progress in understanding the cellular and molecular basis of viral pathogenesis has come from animal models. Like for all modeling approaches that use surrogates, one main limitation concerns the validity of extrapolating data derived from specific experiments using animal models to the general human clinical conditions. Nevertheless, it should be emphasized that animal models have largely participated to the study of viral pathogenesis, and as underlined in this review, they have greatly contributed to delineate main aspects of HTLV-1 pathology. It is not surprising that these models include rabbits, rats, and mice, i.e., three of the main laboratory animals. Their use to study viral pathogenesis has led to complementary data. Indeed, the first two of them – rabbits and rats – can be infected with HTLV-1 and have been used to address essential issues, such as entrance and spread in the host, and the development of the immune response. Conversely, mice that cannot be infected with HTLV-1 have first been instrumental either as immunodeficient strains or as transgenic models. Later on, the generation of humanized mice developing a HIS has generated new hopes in the field of human infectious diseases and represent a physiologically potent system allowing studies of direct interactions of HTLV-1 with human hemato-lymphoid cells. Nevertheless, the present survey of the literature devoted to animal models of HTLV-1 infection and pathogenesis has underlined the specificity as well as the complementarity of each of these models.

## Conflict of Interest Statement

The authors declare that the research was conducted in the absence of any commercial or financial relationships that could be construed as a potential conflict of interest.
